# Calcium phosphate cement with icariin-loaded gelatin microspheres as a local drug delivery system for bone regeneration

**DOI:** 10.1186/s12938-022-01052-0

**Published:** 2022-12-22

**Authors:** Ning Liu, Shuo Huang, Fang Guo, Shafei Zhai, Danyang Wang, Fang Li, Changkui Liu

**Affiliations:** 1 Department of Oral and Maxillofacial Surgery, School of Stomatology, Xi’an Medical University, Xi’an, 710021 Shaanxi China; 2 Department of Oral Histopathology, School of Stomatology, Xi’an Medical University, Xi’an, 710021 Shaanxi China; 3 Department of Prosthodontics, School of Stomatology, Xi’an Medical University, Xi’an, 710021 Shaanxi China; 4 State Key Laboratory of Military Stomatology & National Clinical Research Center for Oral Diseases & Shaanxi Key Laboratory of Stomatology, Department of Prosthodontics, School of Stomatology, Air Force Military Medical University, Xi’an, 710032 Shaanxi China

**Keywords:** Icariin, Gelatin microspheres, Calcium phosphate cement, Controlled release, Bone regeneration

## Abstract

**Background:**

Icariin (ICA), a main active ingredient of Herba Epimedium, could promote bone formation, inhibit bone resorption and alleviate inflammatory responses. The aim of this study was to investigate the effect of ICA on the inhibition of bacteria associated with peri-implantitis, and fabricate a calcium phosphate cement (CPC) with ICA-loaded gelatin microspheres (GMs) as a local drug delivery system efficiently promoting bone formation and alleviating inflammation.

**Results:**

In this study, ICA exhibited antibacterial activity against *P. gingivalis* with a MIC value of 1 × 10^–4^ mol/L. When the concentration of ICA was 0.5 mM, the encapsulation efficiency of GMs reached the maximum value of 76.26 ± 3.97%. GMs with ICA revealed a controlled release profile, 0.5 mM ICA exhibited a higher ICA release profile than the other groups during a 21 d monitoring span. The results of SEM and XRD demonstrated successful fabrication of a calcium phosphate cement with ICA-loaded GMs. ICA released from CPC/GMs (ICA) was slower than ICA released from GMs within 10 days. CPC/GMs (ICA) exhibited antibacterial activity against *P. gingivalis*, but the antibacterial rate of CPC/GMs (ICA) was only 17.15 ± 6.06%. In addition, CPC/GMs (ICA) promoted the proliferation of BMSCs and significantly stimulated the differentiation and maturation of BMSCs. In vivo, H&E and Masson staining experiments demonstrated that CPC/GMs (ICA) exhibited better capacity for bone regeneration than CPC/GMs and CPC, and the expression of TNF-α and IL-1β in the tissue around CPC/GMs (ICA) was significantly lower than CPC/GMs and CPC in IHC staining (*P* < 0.05).

**Conclusion:**

In this study, ICA exhibited limited antibacterial activity against bacteria associated with peri-implantitis. A composite material of calcium phosphate cement with ICA-loaded gelatin microspheres was developed, which not only promoting osteoinductivity and bone formation, but also alleviating inflammation, demonstrating its potential as a promising bone substitute material for treatment of peri-implantitis.

## Background

Dental implants have become an indispensable treatment for patients who are in need to replace missing teeth, with an estimated survival rates of implants supporting fixed dental prostheses of 95.6% after 5 years and 93.1% after 10 years [1]. However, peri-implantitis is considered the most challenging biological complication. Similar to periodontitis, the primary etiologic reason for the inflammation of peri-implant tissues is the oral biofilm [2]. In most cases the composition of the biofilm is similar to the subgingival biofilm of chronic periodontitis that is dominated by Gram-negative bacteria, such as *Porphyromonas gingivalis*, *Fusobacterium nucleatum*, *Prevotella intermedia* and *Aggregatibacter actinomycetemcomitans* [3]. Untreated peri-implantitis can lead to bone resorption and result in implant loss [4].

In purpose of restore bone tissue, various approaches have been recommended. The gold standard for bone reconstruction is the autologous bone graft, but this technique is limited for lack sources of bone, morbidity of the donor site and risk of infection [5–7]. Alternatively, the application of biomaterials in combination with resorbable membranes has become a major treatment option for bone regeneration. A large amount of biomaterials have been used including deproteinized bovine bone, polymers, bioactive glasses and calcium phosphate cements (CPCs), et al. [8–11]. Among them, CPCs are clinically applied in dentistry and orthopedics due to their favorable compatibility to bone tissue [11]. Besides the potential to mimic the mineral phase of bone, CPCs could be injected to perfectly fill the irregularly shaped bone defects and implant sites, then harden within minutes in situ at room temperature. A disadvantage of CPCs is the high stability with slow degradation over time [12]. Ideally the hardened CPCs should have a suitable composition and adequate porosity to be degraded and replaced by host tissue. Incorporation of gelatin microspheres (GMs) in CPCs has proven to introduce macropores during in situ degradation of the microspheres without unacceptably affecting the handling properties or cement setting [13]. Additionally, these microspheres can be used as drug or biological molecule delivery vesicles for local therapy.

Numerous growth factors have been extensively studied for bone regeneration, such as bone morphogenetic proteins (BMPs), fibroblast growth factors (FGFs), insulin-like growth factors (IGFs) and platelet-derived growth factor (PDGF) [14–17]. However, the high cost and rapid degradation of such expensive growth factors limit their widespread usage in clinics. Therefore, specific traditional Chinese medicine, which can promote osteogenesis, is an alternative drug with a high level of production and low cost. Icariin (ICA) is the main pharmacological component of Herba Epimedium, which is a centuries-old traditional herb medicine. Evidence is steadily demonstrating that ICA may play a dual role in bone health by stimulating bone formation while simultaneously inhibiting bone resorption [18]. In addition, ICA could alleviate the inflammatory responses and regulate the immune reaction, it has been reported that local injection of ICA promoted periodontal tissue regeneration and exerted anti-inflammatory and immunomodulatory functions in a minipig model of periodontitis [19]. Furthermore, ICA can be steadily and locally released from biomaterials, making it an attractive osteoinductive candidate for bone tissue engineering [20]. However, to the best of our knowledge, there are few researches on the inhibitory effect of ICA against anaerobic bacteria associated with peri-implantitis.

The aim of this study was to investigate the effect of ICA on the inhibition of bacteria associated with peri-implantitis, and fabricate a novel calcium phosphate cement with ICA-loaded gelatin microspheres as a local drug delivery system efficiently promoting bone formation and alleviating inflammation. The physicochemical characteristics, biocompatibility, osteoinductivity, bone formation and inflammatory reaction of the cements were investigated.

## Results

### Antibacterial activity of ICA

Remarkably, ICA in solution displayed antimicrobial activity at high concentrations (MIC values above 1 × 10^–4^ mol/L) against *P. gingivalis* (Table 1). However, ICA was devoid of significant antimicrobial activity against *F. nucleatum*. MBC value of ICA was not found in the concentration range from 1 × 10^–9^ mol/L to 1 × 10^–2^ mol/L of the bacterial species tested.

**Table 1:** MIC and MBC values for ICA against *P. gingivalis* and *F. nucleatum*

Microorganisms	MIC (mol/L)	MBC(mol/L)
*P. gingivalis*	1 × 10^–4^	–
*F. nucleatum*	–	–

### Encapsulation efficiency

It can be seen that the amount of ICA had a significant effect on encapsulation efficiency of gelatin microspheres (Table 2). The encapsulation efficiency of microspheres was variable from 68.48 ± 3.41% to 76.26 ± 3.97%. When the concentration of ICA was 0.5 mM, the encapsulation efficiency reached the maximum value of 76.26 ± 3.97%.

**Table 2:** Encapsulation efficiency of gelatin microspheres

	GM-1	GM-2	GM-3	GM-4
ICA concentration (mM)	0.25	0.5	1	2
Encapsulation efficiency (%)	68.48 ± 3.41	76.26 ± 3.97	75.32 ± 0.92	75.2 ± 1.14

### In vitro release test

**Fig.1:**
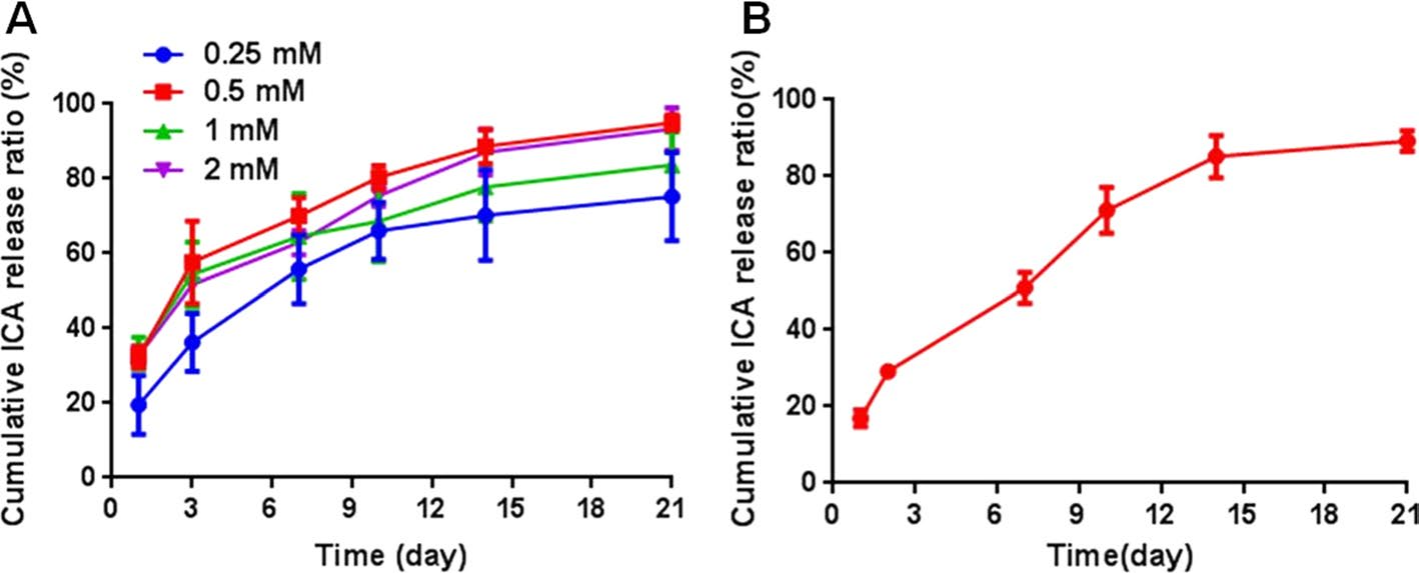
In vitro release profile of ICA:** A** ICA released from gelatin microspheres,** B** 0.5 mM ICA released from cement

As shown in Fig. 1A, a sustained release profile was observed in ICA-loaded GMs during the 21 days period. The cumulative ICA release ratio in the various ICA concentrations displayed the same general trend. Approximately 30% of the total initial quantity was released after 24 h, a rapid initial release by 10 days followed by a slow release, reaching equilibrium at day 21. When the release test was carried out to 21 days, 0.5 mM ICA-loaded GMs exhibited a higher ICA release profile than the other groups, the cumulative ICA release percentage was reached to 95.06 ± 2.05%. The cumulative ICA release ratio of the cement was shown in Fig. 1B, ICA released from CPC/GMs (ICA) was slower than ICA released from GMs within 10 days. When the release test was carried out to 21 days, the cumulative ICA release percentage of the cement was reached to 89.21 ± 2.68%.

### Morphology of GMs and cements

**Fig.2:**
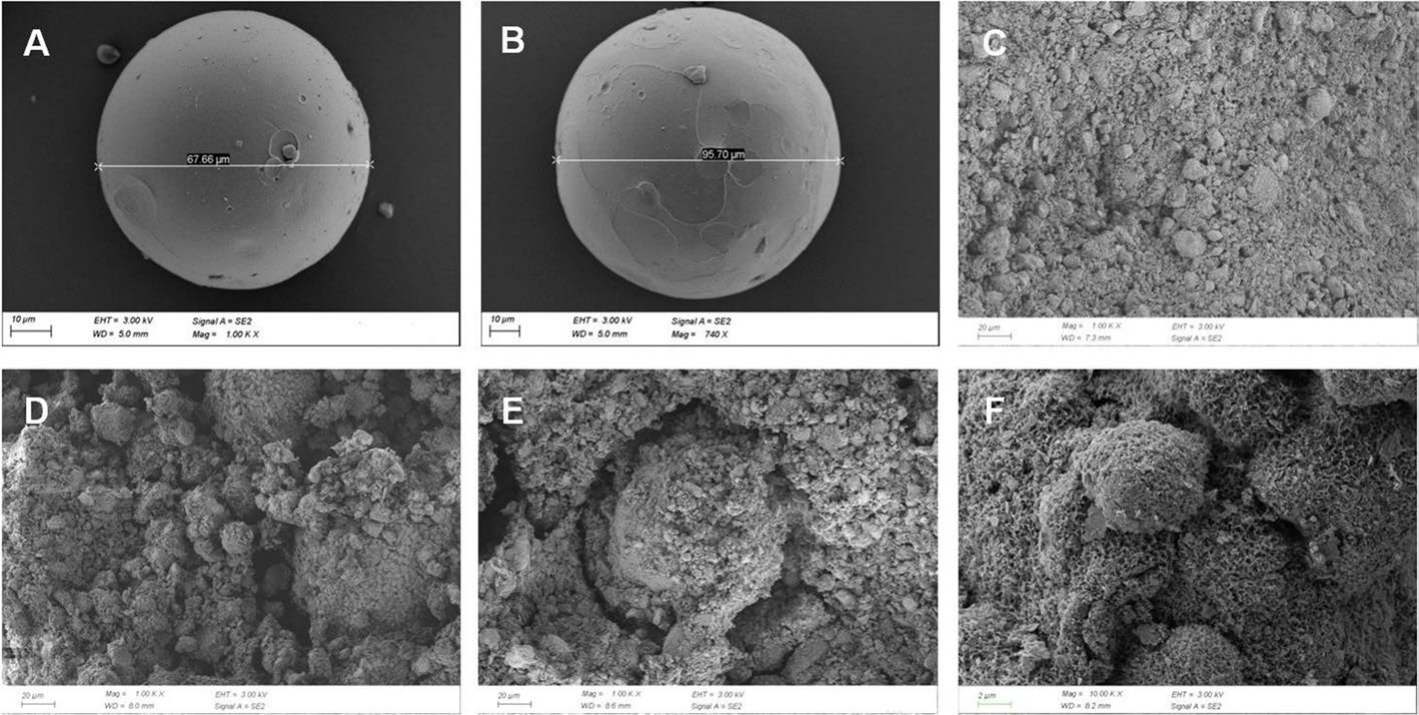
SEM images of the morphology: **A** GMs, **B** GMs loaded with 0.5 mM ICA, **C** CPC, **D** CPC/GMs, **E** CPC/GMs(ICA) and **F** CPC/GMs(ICA) after 1 week of soaking

The morphologies of the gelatin microspheres with or without 0.5 mmol/L icariin showed well-preserved, rounded particles, and the diameters of gelatin microspheres were ranging from 10 to 110 μm (Fig. 2A, B). SEM image of the set CPC showed a typical coarse structure (Fig. 2C). However, CPC/GMs and CPC/GMs(ICA) displayed a tight packing of the gelatin microspheres, gelatin microspheres were dispersed randomly in the CPC matrix, with most of them being covered with a thin layer of CPC matrix, indicating that the gelatin microspheres with or without 0.5 mM ICA had good adhesion with the CPC matrix (Fig. 2D, E). After 1 week of soaking, a matrix of small apatite-like crystals could be seen in the CPC/GMs (ICA) sample (Fig. 2F).

### X-ray diffraction analysis

**Fig.3:**
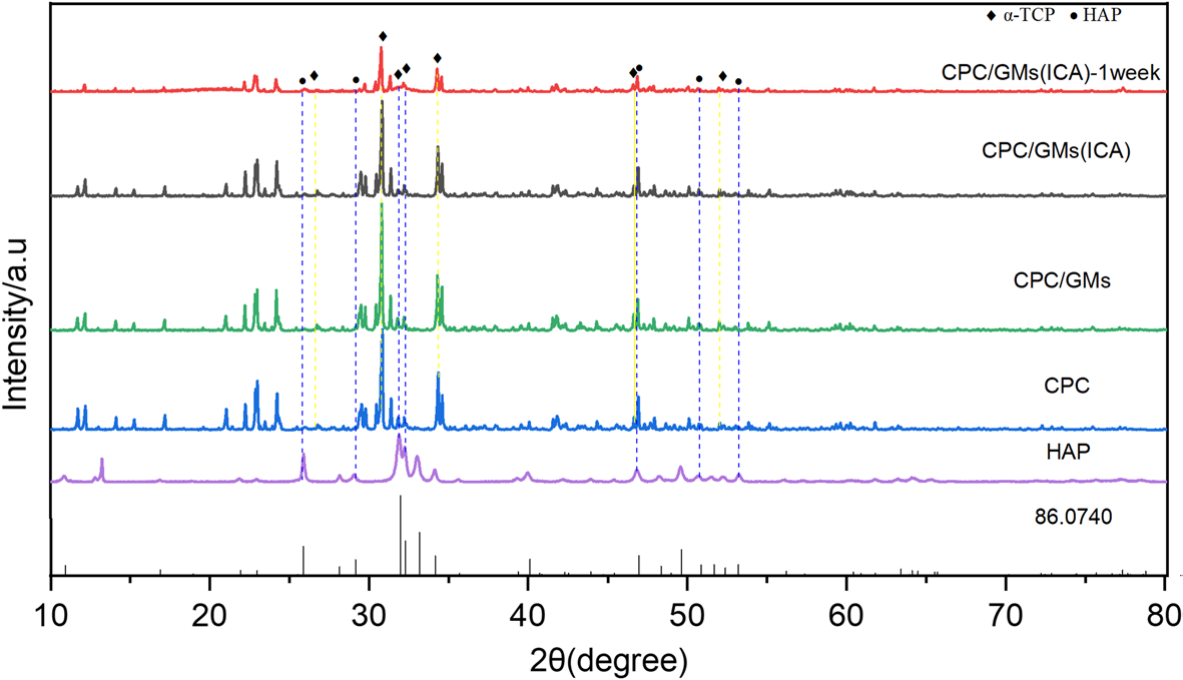
XRD spectra of the cements

As shown in Fig. 3, the XRD patterns of CPC/GMs and CPC/GMs (ICA) are similar to that of original CPC, which mainly consisted of two components alpha-tricalcium phosphate (α-TCP) and hydroxyapatite (HAP), unaltered by inclusion of 10 wt% GMs with or without 0.5 mM ICA. After 1 week of soaking in PBS, the hydration products of CPC/GMs (ICA) were remain consistent with those of the original CPC. However, the diffraction peaks related to α-TCP became weak, resulting into a phase transformation of α-TCP to crystalline HAP.

### In vitro antibacterial property

**Fig.4:**
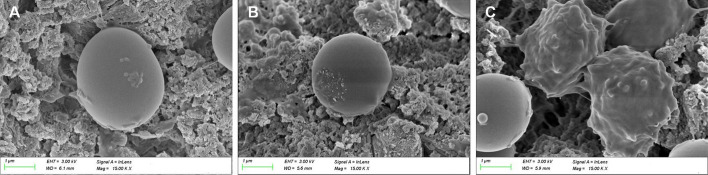
Morphologies of *P. gingivalis* by SEM after 24 h of culture on the cements: **A** CPC, **B** CPC/GMs, **C** CPC/GMs(ICA)

**Fig.5:**
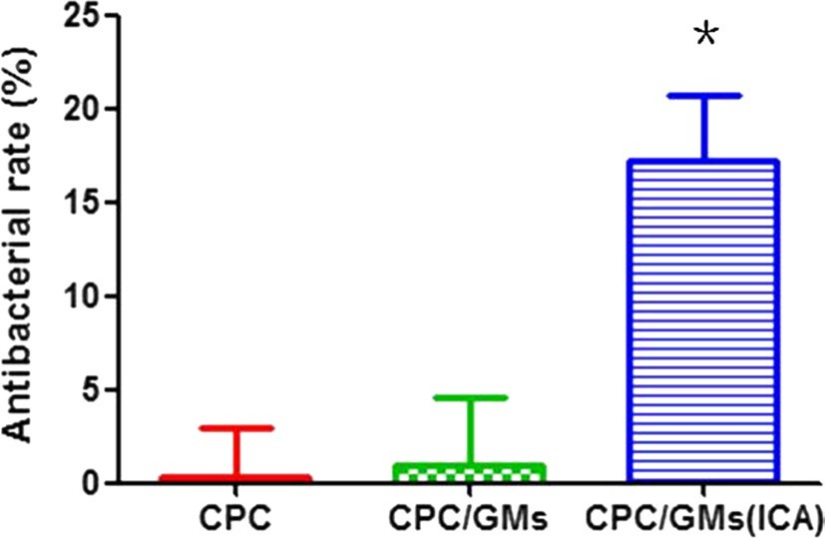
Antibacterial rates of the cements against *P. gingivalis*

SEM images were shown in Fig. 4. *P. gingivalis* on CPC and CPC/GMs were alive with a smooth spherical morphology (Fig. 4A, B). Some bacteria on CPC/GMs (ICA) were dead with a anomalous morphology. While, Some bacteria were still alive with a smooth spherical morphology (Fig. 4C). The results of bacterial counting assay showed that the antibacterial rate of CPC/GMs(ICA) was 17.15 ± 6.06%, significantly higher than CPC and CPC/GMs (*P* < 0.05) (Fig. 5).

### Cell proliferation

**Fig.6:**
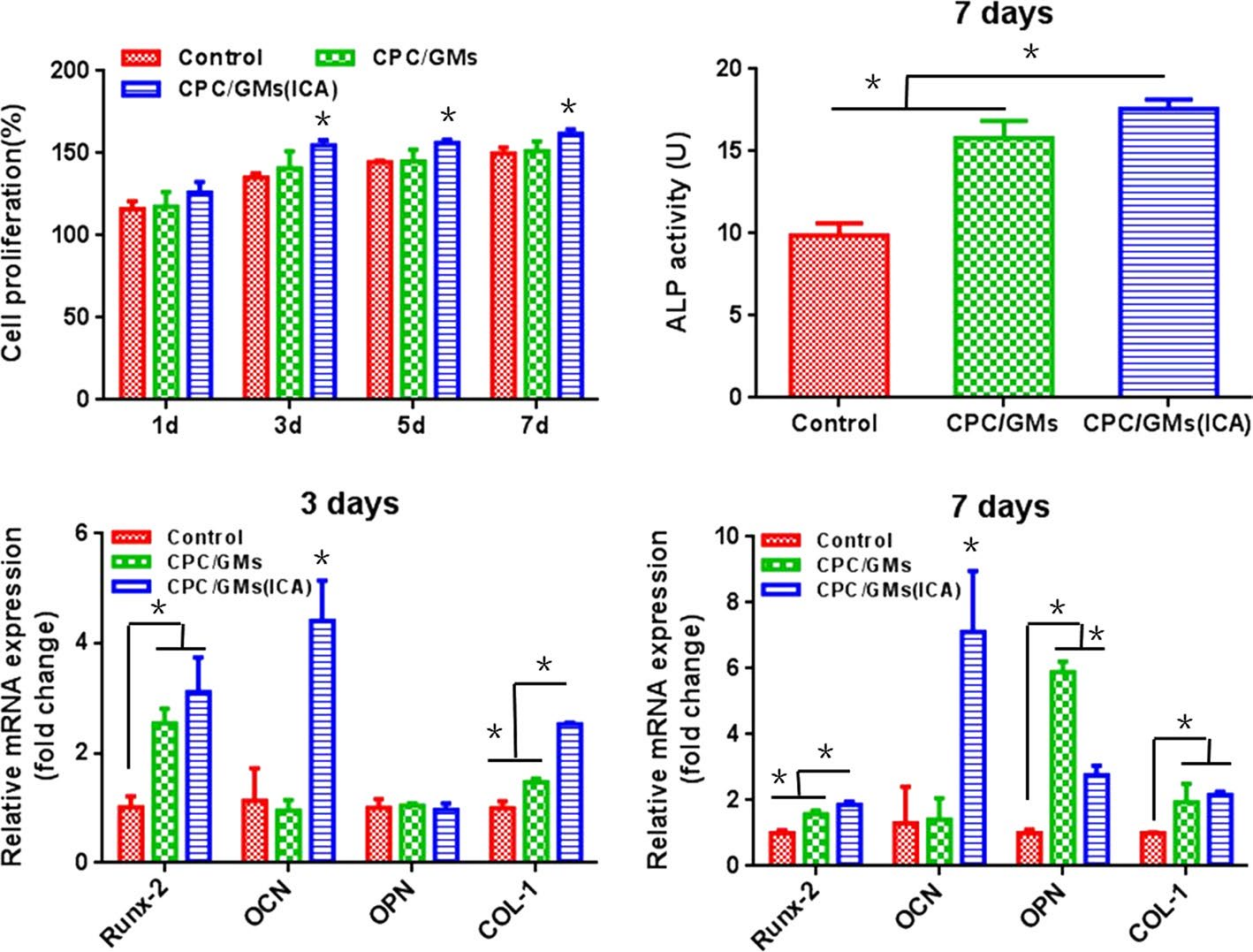
**A** The cell proliferation from 1 to 7 d by CCK-8; **B** Alkaline phosphatase activity of BMSCs incubation for 7 days; **C**, **D** Expression of typical osteogenic genes during the in vitro culture for 3 and 7 days. *denotes significant difference (*P* < 0.05)

The CCK-8 assay was performed on 1, 3, 5 and 7 d to evaluate the proliferation ability of BMSCs cultured with leaching liquors (Fig. 6A). CPC/GMs did not affect cell proliferation of BMSCs compared with the control group. However, CPC/GMs (ICA) group significantly increased the cell proliferation on 3, 5 and 7 d compared with CPC/GMs and the control group but not on the first day (*P* < 0.05).

### Alkaline phosphatase activity

ALP can be used as an early marker of osteogenic differentiation. The ALP activity of BMSCs cultured for 7 days was shown in Fig. 6B. The CPC/GMs group had higher ALP activity than the control group (*P* < 0.05). In addition, higher ALP activity was produced by CPC/GMs (ICA) group than the CPC/GMs group and the control group (*P* < 0.05).

### Gene expression analysis

The gene expression of Runx-2, OCN, OPN and COL-1 of BMSCs after 3 and 7 days of culture were evaluated by RT-PCR (Fig. 6C, D). At day 3, the CPC/GMs and CPC/GMs (ICA) groups significantly increased the Runx-2 mRNA expression compared with the control group. The CPC/GMs (ICA) group significantly increased the expression of OCN and COL-1 than CPC/GMs and the control groups. Noticeably, OCN mRNA expression was dramatically promoted by the CPC/GMs (ICA) group. No statistical difference in gene expression of OPN was observed between the three groups. After cultured for 7 days, similar trends in Runx-2, OCN and COL-1 expression of BMSCs were observed. The expression levels of Runx-2 and OCN was highest in the CPC/GMs (ICA) group compared with other groups. The CPC/GMs and CPC/GMs (ICA) groups significantly increased the expression of COL-1 compared with the control group. However, CPC/GMs had significantly higher OPN mRNA expression compared to CPC/GMs (ICA) and the control groups.

### Alizarin Red staining for mineralization

**Fig.7:**
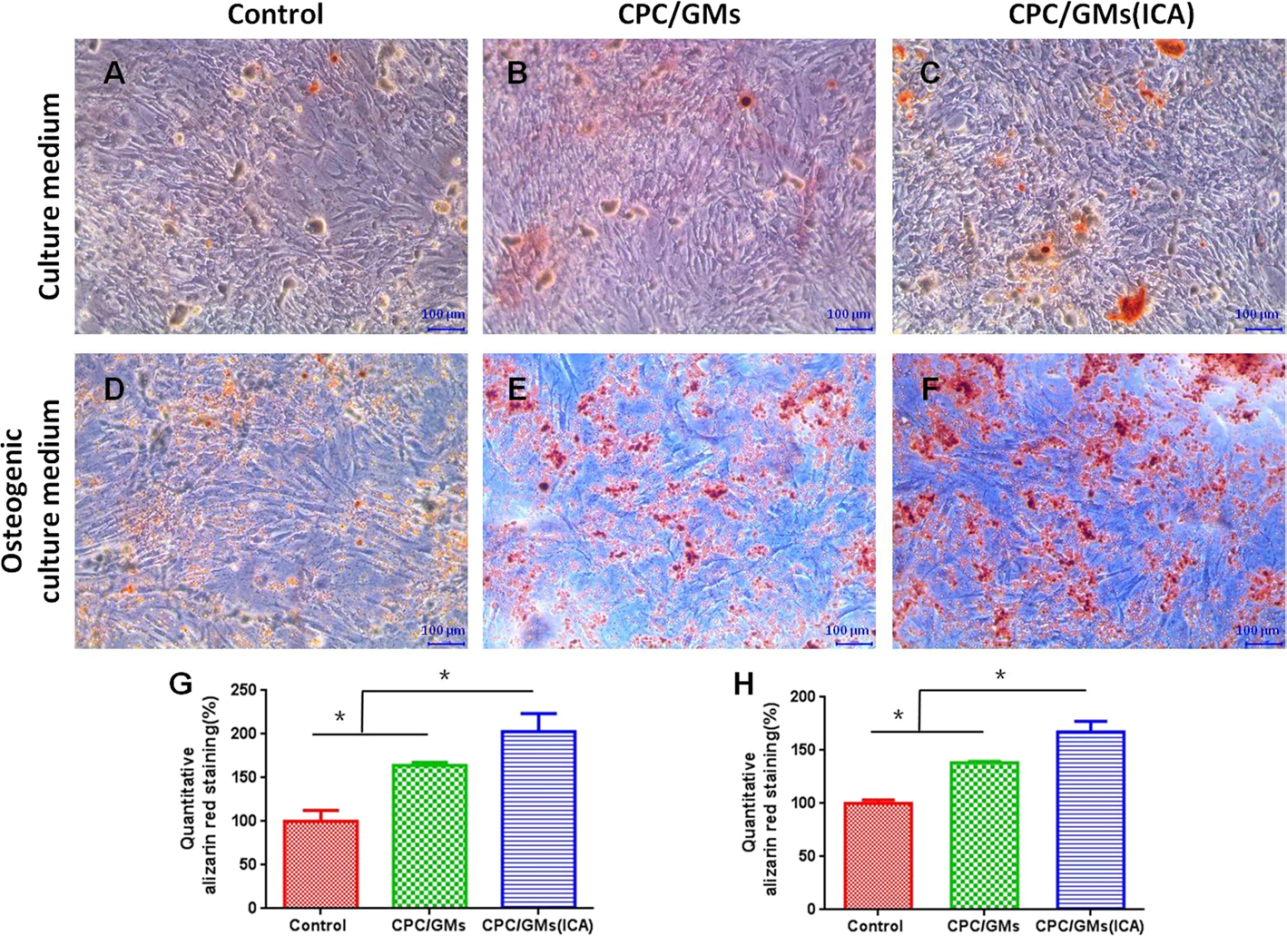
Alizarin Red staining performed to examine extracellular mineralization: **A**, **D** control group, **B**, **E** CPC/GMs group, **C**, **F** CPC/GMs(ICA) group and semi-quantitative analysis of calcium deposition: (G) BMSCs cultured by culture medium or (H) osteogenic culture mediumcontaining 10% (v/v) extracts of cements. *denotes significant difference (*P* < 0.05)

Similar trend was observed in Alizarin Red staining of BMSCs cultured by the culture medium or the osteogenic culture medium containing 10% (v/v) extracts of cements (Fig. 7). After 21 days, BMSCs cultured in CPC/GMs (ICA) extracted medium exhibited more mineralized nodules than in CPC/GMs and the control groups. The CPC/GMs group showed more areas of mineralized nodules compared with the control group. In addition, the results of quantification of mineralization showed that the CPC/GMs (ICA) group had a significant higher calcium deposition compared with CPC/GMs and the control groups (*P* < 0.05).

### Histomorphometric analysis

**Fig.8:**
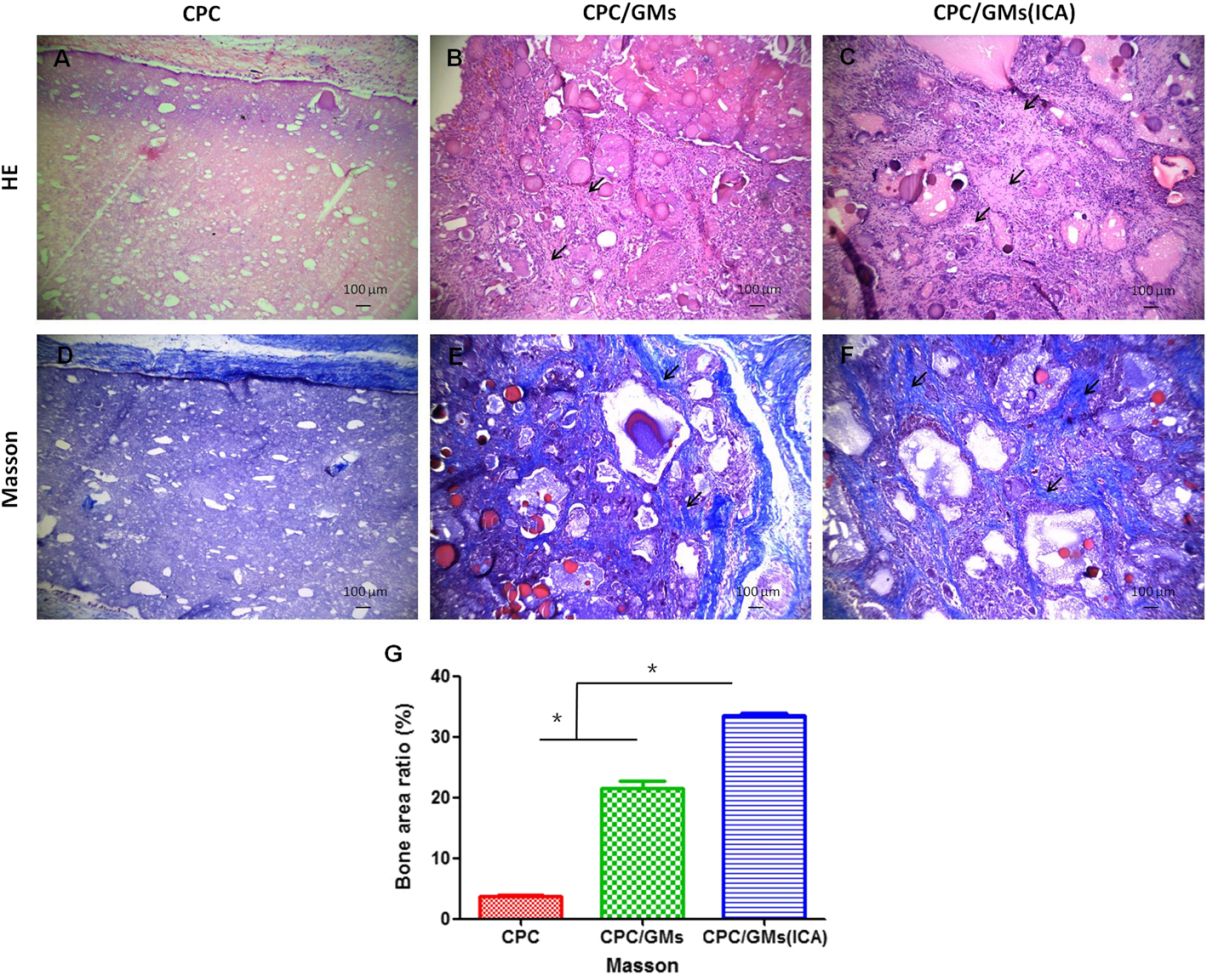
H&E **A**–**C** and Masson **D**–**F** staining of samples at 4 weeks (10 × magnification): **A**, **D** CPC group, **B**, **E** CPC/GMs group, **C**, **F** CPC/GMs(ICA) group, **G** the statistical comparison of Masson staining images. Black arrows indicate new bone. *denotes significant difference (*P* < 0.05)

H&E and Masson staining displayed new bone formation in the three groups after 4 weeks implantation in Fig. 8. In H&E staining, the new bone formation in the CPC/GMs (ICA) group was more evident than the CPC/GMs group, but this formation was not evident in the control group. The in-growth of new blood vessels and residual nondegraded GMs were observed in the CPC/GMs group and CPC/GMs (ICA) group. Similar trend was observed in Masson staining, with larger amount of newly formed collagen tissue in CPC/GMs (ICA) group than in CPC/GMs group and CPC group. The statistical analysis of bone area ration in Masson staining confirmed that the bone-forming ability of CPC/GMs (ICA) group was significantly highest.

### IHC analysis

**Fig.9:**
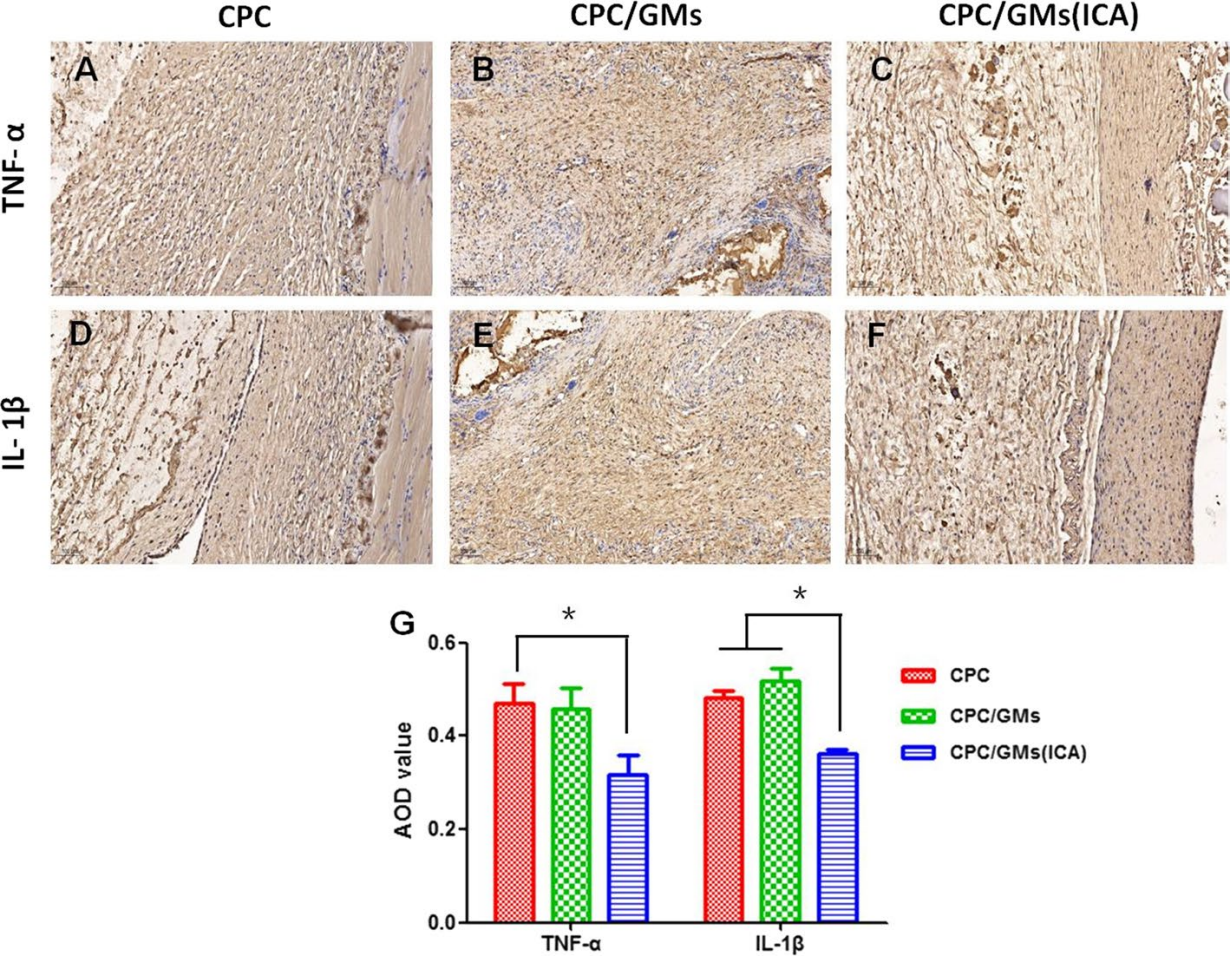
IHC staining and the corresponding quantitative analysis of TNF-α and IL-1β at 4 weeks (10 × magnification): **A**, **D** CPC group, **B**, **E** CPC/GMs group, **C**, **F** CPC/GMs(ICA) group, **G** the corresponding quantitative analysis of TNF-α and IL-1β. *denotes significant difference (*P* < 0.05)

As shown in Fig. 9, the expression of TNF-α from the tissues surrounding the CPC/GMs (ICA) material was significantly decreased, when compared to the CPC (*P* < 0.05). In addition, the expression of IL-1β in the tissue around CPC/GMs (ICA) material was significantly lower than CPC/GMs and CPC (*P* < 0.05). The CPC/GMs (ICA) showed the lowest inflammatory effect among the three groups.

## Discussion

Peri-implantitis is one of the major causes of implant failure in clinic [21]. ICA, a major active constituent in Herba Epimedium, showed to prevent osteoporosis, promote bone formation and alleviate inflammatory responses [18]. However, to the best of our knowledge, there are few researches on the inhibitory effect of ICA against anaerobic bacteria associated with peri-implantitis. In this study, ICA exhibited antibacterial activity against *P. gingivalis* with a MIC value of 1 × 10^–4^ mol/L. On the contrary, ICA could not inhibite *F. nucleatum* proliferation in the concentration range from 1 × 10^–9^ mol/L to 1 × 10^–2^ mol/L. MBC value of ICA was not found within the tested concentrations. Thus, the *P. gingivalis* was selected to study the in vitro antibacterial property of the cements. The results showed that calcium phosphate cement with icariin loaded gelatin microspheres exhibited antibacterial activity against *P. gingivalis*, but the antibacterial rate of CPC/GMs (ICA) was only 17.15 ± 6.06%, which could not achieve satisfactory clinical requirements. However, it was reported that ICA could enhance pre-osteoblast proliferation and differentiation, which could be used as a potential agent for osteogenesis [22–24].

CPCs have been one of the most studied materials due to their favorable biocompatibility, osteoconductivity and moldability [11]. Nevertheless, the drawbacks of CPCs are slow degradability and lack of the property of osteoinductivity, which negatively influence their clinical performance [12]. Gelatin, which has excellent biocompatibility, can accelerate the setting reaction and strengthen the mechanical property of CPCs [25]. Gelatin microspheres were used as a local drug delivery system to improve the osteoinductive property of CPCs in this study. It has been reported that macropore size of similar to 100 μm is beneficial in facilitating cell migration, bone ingrowth and internal mineralized bone formation [22]. The SEM images exhibited that gelatin microspheres with or without 0.5 mmol/L icariin were well-preserved and rounded particles, and the diameters of gelatin microspheres were ranging from 10 to 110 μm. The release kinetics is a key factor for the bioactive efficiency of materials. As a type of drug delivery material for ICA, GMs exhibited a controlled release of ICA in vitro. In this study, the cumulative release amount curve of GMs (ICA) displayed a burst release of ICA within the first 24 h, which can be the result of ICA distributed on the surface of the microspheres readily dissolved in PBS. After this time period, the cumulative release amount curve of CPC/GMs (ICA) displayed a sustained release profile and lasted more than 21 days. It was speculated that the sustained release was probably due to the diffusion of ICA from the interior region of GMs and the degradation of GMs, the concentration gradient providing the driving force. Then, the cumulative ICA release ratio reached an equilibrium state. Based on the results of encapsulation rate and release profile of ICA, GMs with 0.5 mM ICA was chosen to fabricate the composite CPC/GMs (ICA) material. ICA released from CPC/GMs (ICA) was slower than ICA released from GMs within 10 days. It was speculated that the CPC may impeded the diffusion of ICA from the interior of the cement. The SEM images of the developed CPC/GMs and CPC/GMs (ICA) materials showed that gelatin microspheres were dispersed randomly in the CPC matrix, with most of them being covered with a thin layer of CPC matrix, indicating that the gelatin microspheres had good adhesion with the CPC matrix. After 1 week of soaking, a matrix of small apatite-like crystals could be seen, indicating that small amount of new HAP phase formed on the composite cement surface, which was in agreement with the results of the XRD study.

BMSCs are multifunctional stem cells in bone marrow, which can be induced to differentiate into osteoblasts. The biocompatibility of the composite cements was assessed by treating BMSCs in vitro with the leaching liquors containing the released ICA and the degradation products. CCK-8 assay was performed on days 1, 3, 5 and 7. It was observed the cells cultured with the leaching liquors of CPC/GMs (ICA) displayed slightly higher proliferation ability than CPC/GMs and the control groups, indicating that the cements were highly compatible and the cement with ICA-loaded GMs was able to increase BMSCs proliferation. Several studies showed that ICA significantly promoted the proliferation of BMSCs at a concentration range between 10^–9^ and 10^–5^ mol/L, although the optimal concentration remains uncertain [26, 27]. It was reasonable to presume that the concentration of ICA loaded in the calcium phosphate cements was appropriate for further experiments.

To evaluate the osteogenicity of the composite cements, the expressions of some osteogenic specific factors were determined. ALP is a calcium-binding transporter that promotes cell maturation and calcification, and can be used as one of the early markers of osteogenic differentiation. The expression of ALP was particularly high in the CPC/GMs(ICA) group on day 7, indicating that the leached extract of the ICA-loaded cement would improve the osteogenic differentiation of BMSCs. In addition to the overexpression of ALP, BMSCs also synthesized and secreted a large number of bone matrix substituents during osteogenic differentiation. Previous studies showed that ICA stimulates the osteogenic differentiation of BMSCs into osteoblasts through multiple signaling pathways, including BMP (bone morphogenetic protein), MAPK (mitogen-activated protein kinase), NO (nitric oxide), and the canonical Wnt/β-catenin pathways [28]. In this study, the expressions of Runx-2, OCN and COL-1 in the CPC/GMs(ICA) group were higher than that in CPC/GMs and the control groups on days 3 and 7, indicating that the leaching liquor of CPC/GMs(ICA) could upregulate the expression of osteogenic differentiation-related genes in the presence of ICA. In addition, the expression of Runx-2, OPN and COL-1 in CPC/GMs group was higher than those in the control group on day 7, indicating the leaching liquor of CPC/GMs also increased osteoblast differentiation. Recent studies showed that ICA loaded in scaffolds could slowly release ICA in response to BMSCs, thereby augmenting their rate of osteoblast differentiation [20, 22, 29]. Similar to previous studies, our study revealed that the incorporation of ICA in calcium phosphate cement had appreciable effects on BMSCs osteogenic differentiation.

Mineralization is very important for bone regeneration. It was reported that ICA alone could increased the in vitro mineralization [23]. In this study, the results showed that the leaching liquor of CPC/GMs was effective for promoting mineralization on BMSCs in vitro, probably due to calcium phosphate cement serving as calcium and phosphate ion sources. Besides, the addition of ICA in CPC/GMs significantly enhanced the mineralization ability as expected in this study. The in vivo results of the present study provided further validation for the superiority of CPC/GMs (ICA) in bone formation. The outcome of histological analysis showed that CPC/GMs (ICA) induced more new bone formation than CPC/GMs and CPC, indicating that incorporating ICA in CPC/GMs might not only improve the in vitro osteogenic differentiation and mineralization of stem cells, but also enhance in vivo bone fomation, which has considerable potential for use as a drug-loaded biomaterial for bone repair.

Peri-implantitis is a polymicrobial anaerobic infection and inflammatory disease. TNF-α plays an important role in the pathogenesis of peri-implantitis and is involved at an early stage in the inflammatory cascade to promote the release of other inflammatory mediators to amplify inflammation [30]. IL-1β is frequently detected in overt inflamed gingival tissues, affecting the biological activities of connective tissue cells and exerting a catabolic effect on bone [31]. A recent study reported that local injection of ICA promoted periodontal tissue regeneration and exerted anti-inflammatory and immunomodulatory functions in a minipig model of periodontitis [17]. The present study showed that the expression of TNF-α and IL-1β in the tissue around CPC/GMs(ICA) material was significantly lower than CPC/GMs and CPC in IHC staining (*P* < 0.05), demonstrating the calcium phosphate cement loaded with icariin alleviated inflammation after operation in the backs of rabbits. Further study with large animal model and longer implantation period will be investigated in future.

## Conclusions

In this study, ICA exhibited limited antibacterial activity against bacteria associated with peri-implantitis. A novel composite material of calcium phosphate cement with ICA-loaded gelatin microspheres was developed, which not only promoting osteoinductivity and bone formation, but also alleviating inflammation, demonstrating its potential as a promising bone substitute material for treatment of peri-implantitis.

## Materials and methods

### Bacteria culture

*Porphyromonas gingivalis* (*P. gingivalis*, ATCC 33,277) and *Fusobacterium nucleatum* (*F. nucleatum*, ATCC 10,953) were cultivated in freshly prepared brain heart infusion (BHI) agar plate and supplemented with 5% sterile defibrinated sheep blood, 1% hemin and 0.1% menadione (MilliporeSigma, USA) in a chamber under anaerobic conditions of 80% N_2,_ 10% H_2_ and 10% CO_2_ at 37 °C.

### Determination of minimal inhibitory concentration (MIC) and minimal bactericidal concentration (MBC)

The inhibition effect of ICA in the presence of different concentrations was determined according to broth microdilution antibacterial assay. In short, the bacteria inoculum (*P. gingivalis* and *F. nucleatum*), allowed to grow exponential phase, were incubated with ICA to determine MIC. A volume of 100 μL of bacterial culture (2 × 10^6^ CFU/mL) and 100 μL of ICA solutions at final concentrations of 1 × 10^–9^ to 1 × 10^–2^ mol/L were placed into 96-well plates and incubated at 37 °C for 48 h. The MIC was taken as the concentration of ICA at which no microbial growth was observed with the unaided eye visually and the spectrophotometric method via optical density measurement at 600 nm (Epoch2, BioTdk, USA). MBC values were assessed by plating in BHI agar plate the content of the wells where no visible growth was observed.

### Preparation of ICA-loaded GMs

GMs were prepared by using W/O emulsion chemically crosslinking method. Briefly, 3 g gelatin was dissolved in 30 mL distilled water at 40 °C to get 10 wt.% gelatin solution, then 0.005, 0.010, 0.020 and 0.040 g ICA was added into the gelatin solution, the final concentration of ICA was 0.25, 0.5, 1 and 2 mM. The resulting solution dripped slowly into 300 mL vegetable oil at 50 °C. The biphasic system was thoroughly mixed to form a W/O emulsion using a propeller at 450 rpm for about 60 min. Subsequently, the stirring was continued in the ice bath so that the temperature of oil phase was kept at 4 °C. After 60 min, microspheres were cross-linked for 15 min using 50% glutaraldehyde solution, collected by filtration, then rinsed with acetone and ethanol to remove the remaining oil on their surfaces. Finally, the dried GMs were stored for further use.

### Encapsulation efficiency

The amount of gelatin microspheres (0.2 g) respectively containing 0.25, 0.5, 1 and 2 mM ICA were treated with 1 mol/L sodium hydroxide to fully dissolve microspheres. Then, the solution was adjusted to neutral solution and centrifugated for 10 min, the supernatant was mixed with methanol to the volume of 10 mL. The absorbance of the solution was measured by monitoring absorbance at 270 nm. The measured absorbance was then converted to the amount of ICA by using standard calibration curve. The encapsulation efficiency was calculated as follows:

$$ {\text{Encapsulation efficiency }}\% \, = {\text{ Entrapped amount of drug }}/{\text{ Theoratical amount of drug }} \times { 1}00\% $$

### In vitro ICA released from gelatin microspheres

The release profiles of ICA from gelatin microspheres were carried out. Briefly, 0.2 g gelatin microspheres with 0.25, 0.5, 1 and 2 mM ICA were immersed in 5 mL of phosphate-buffered saline (PBS) solution and gently shaken at 100 rpm at 37 °C. At predetermined time points of 1, 3, 7, 10, 14 and 21 days, samples were centrifuged at 3000 rpm for 5 min, all supernatants were collected and fresh PBS was replaced, samples were stored at 4 °C. For analysis of ICA concentrations, solutions were measured by monitoring absorbance at 270 nm. The release of ICA from gelatin microspheres was calculated according to the standard curve, and the percentage of ICA released was calculated by dividing the cumulated amount of released drug by the total amount of drug loaded.

### Preparation of the composite cements

The composite cement powders, consisted of 90 wt% the plain cement (86 wt% α-TCP, 5 wt% CaCO_3_, 5 wt% CaHPO_4_·H_2_O and 4 wt% HAP) and 10 wt% GMs, were mixed to prepare the composite cements. The GMs loaded with 0.5 mM ICA was used as an experimental group, GMs alone was used as control group. The aqueous solution of 2.5 wt% Na_2_HPO_4_ was used as the liquid part, the composite cement specimens were prepared by using a liquid-to-powder (L/P) ratio of 0.4 ml/g. After mixing, the pastes were placed into molds to form specimens.

### Characterization of GMs and cements

The morphology of GMs and cements was observed by scanning electron microscopy (Carl Zeiss, Germany) at an accelerating voltage of 5 kV after the samples were sputter-coated with gold before observation. The composition of the cements was analyzed by X-ray diffraction (XRD, Bruker, Germany). The cements were grinded into powders and exposed to a CuKα radiation (40 kV, 30 mA). The scan step was 10°/min and a diffraction of 10–80° (2θ) was analyzed.

### ICA released from cement

Three samples of calcium phosphate cement with 0.5 mM ICA were immersed in 5 mL of PBS solution and gently shaken at 100 rpm at 37 °C. At predetermined time points of 1, 2, 7, 10, 14, 21 days, 0.5 mL solution was collected and the same volume of fresh PBS was replaced. The cumulative release ratio of ICA released from cement was calculated as described in the assay of ICA released from gelatin microspheres.

### In vitro antibacterial evaluation

*Porphyromonas gingivalis* (*P. gingivalis*, ATCC 33,277) which has been proved to associate with dental peri-implantitis, was used to investigate the antibacterial property of the icariin loaded calcium phosphate cements. *P. gingivalis* was cultured in freshly prepared brain heart infusion (BHI) agar plate and supplemented with 5% sterile defibrinated sheep blood, 1% hemin and 0.1% menadione in a chamber under anaerobic conditions of 80% N_2,_ 10% H_2_ and 10% CO_2_. After incubation, thirty microliters of bacterial suspension with 1 × 10^7^ CFU/mL bacteria was seeded on six samples of each group. After 24 h of culture under anaerobic conditions at 37 °C, three samples of each group were fixed with 2.5% glutaraldehyde at 4 °C overnight, dehydrated in a series of ethanol, and finally observed using SEM (GeminiSEM 300, Carl Zeiss, Germany). The other three samples of each group were transferred into centrifuge tubes containing 2 mL of BHI. The tubes were agitated intensely to detach the bacteria, then the detached bacteria suspension was diluted 1000 times with BHI. Thirty microliters of diluted bacterial suspension was spread onto agar culture plates. After 3 days of culture, the live bacteria colonies were counted and the antibacterial rate was calculated using the following equation:

$$ {\text{R }}\left( \% \right) \, = \, \left( {{\text{N}}_{0} - {\text{N}}_{{1}} } \right) \, /{\text{ N}}_{0} \times {1}00\% , $$

In which, N_0_ is the average number of the CPC group, and N_1_ represents the average number of the CPC/GMs and CPC/GMs (ICA) groups.

### Cell culture

BMSCs were isolated from femur and tibiae of Sprague Dawley rats (1 weeks old, male) under sterile conditions and then cultured in alpha minimum essential medium (α-MEM, HyClone, USA) supplemented with 10% fetal bovine serum (Gibco, USA) and 1% penicillin/streptomycin (Gibco, USA) at 37 °C under atmosphere conditions of 95% humidity and 5% CO_2_. After 2 days of culture, non-adherent cells were washed away with PBS. The culture medium was refreshed every 3 days, and BMSCs were expanded when 80–90% confluence was reached. All experiments were performed using BMSCs within passage five. The proliferation and osteogenic differentiation experiments were determined by investigating the leached extracts of the cements on BMSCs.

### Preparation the leached extracts of the cements

The cell proliferation and osteogenic differentiation experiments were investigated by culture medium containing the extract liquid of cements on BMSCs. Cements experienced a series of sterilization process. Then, 0.1 g cements were soaking in 10 mL α-MEM, the leaching liquors were extracted at the fixed time points. BMSCs were exposed to α-MEM culture medium containing 10% (v/v) media extracts of CPC/GMs and CPC/GMs loaded with 0.5 mM ICA, normal cell culture medium was used as control.

### Cell proliferation

BMSCs viability and proliferation were determined using a cell counting kit-8 assay (CCK-8, Dojindo, Japan). In brief, BMSCs were seeded in 96-well plates at an initial density of 3 × 10^3^ cells/well in 100 μl culture medium. After 24 h, cells were treated with culture medium containing 10% (v/v) extract liquid of cements, cells in culture medium served as control. At the time point of 1, 3, 5 and 7 d, fresh culture medium containing CCK-8 reagent at a ratio of 10:1 (v/v) was added into each well, and incubated for 2 h at 37 °C. Subsequently, the optical density (OD) values of the supernatants were determined using a microplate reader (BioTek, USA) at a wavelength of 450 nm.

### Alkaline phosphatase activity

Alkaline phosphatase (ALP) activity was determined using a quantitative ALP assay. BMSCs (1 × 10^4^ cells/well) were cultured in a 24-well plate and incubated with culture medium containing 10% (v/v) extract liquid of cements, cells in culture medium served as control. After 7 days of culture, cells were washed three times twice with PBS and then lysed with 0.5 ml of 0.1% Triton X-100 at 37 °C for 30 min, the solutions were centrifuged at 3000 rmp for 5 min. The supernatants were used to determine ALP activity according to the manufacturer's instructions (Jiancheng Technology, China).

### Gene expression analysis

In this experiment, BMSCs were seeded in 6-well plates at a density of 1 × 10^5^ cells per well and incubated with culture medium containing 10% (v/v) extract liquid of cements, cells in culture medium served as control. Gene expression was observed at 3 and 7 days of culture. Total RNA was extracted from each sample using TRIzol reagent (Roche, Switzerland). The reverse transcription was performed with a Transcriptor First Strand cDNA Synthesis Kit (Roche, Switzerland) to produce the required cDNA. Real-time polymerase chain reaction (RT-PCR) was performed for the quantification of gene expression and a FastStart Universal SYBR Green Master (Roche, Switzerland) in a Real-Time PCR System (LightCycler^®^96, Roche, Switzerland). All reactions were carried out in triplicates. The primer sequences of osteogenic genes including runt-related transcription factor 2 (RUNX-2), osteocalcin (OCN), osteopontin (OPN) and collagen type 1 (COL-1) are listed in Table 3. Target genes were normalized against glyceraldehydes-3-phosphatedehydrogenase (GAPDH) expression.

**Table3:** The parameters of primers utilized for detecting osteogenetic gene expression

Gene	Direction	Sequence(5′- 3′)
Runx2	Forward Reverse	TCCGCCACCACTCACTACCAC GGAACTGATAGGACGCTGACGAAG
OCN	Forward Reverse	GGACCCTCTCTCTGCTCACTCTG ACCTTACTGCCCTCCTGCTTGG
OPN	Forward Reverse	GACGATGATGACGACGACGATGAC GTGTGCTGGCAGTGAAGGACTC
COL- 1	Forward Reverse	GCACTGGCGATAGTGGTCCT CCGGTAGTAACGGCCACCAT
GAPDH	Forward Reverse	GACATGCCGCCTGGAGAAAC AGCCCAGGATGCCCTTTAGT

### Alizarin Red S staining for mineralization

Alizarin Red S staining had been used to visualize calcium-rich deposits of cells in culture. BMSCs were seeded in a 24-well plate at an initial density of 0.5 × 10^4^ cells/well in 100 μl culture medium. After 24 h, the culture medium was refreshed with culture medium containing 10% (v/v) extracts of cements or the culture medium containing 10% (v/v) extracts of cements supplemented with 0.25 mM ascorbic acid, 10 mM β-glycerophosphate and 10 nM dexamethasone (MilliporeSigma, USA). On day 21, the samples were washed twice with PBS and fixed in 4% paraformaldehyde for 30 min, and then stained in 1% (w/v) Alizarin Red S solution for 30 min at room temperature. After washing thrice, the plates were photographed under an optical microscope. In the quantitative analysis, the Alizarin Red stain was dissolved in 10% cetylpyridinum chloride (MilliporeSigma, USA) for 30 min. The mixture was placed in 96-well plates and detected by microplate reader at 570 nm for quantification of calcium deposition.

### In vivo study

The in vivo experiments of the composite cements were investigated by implantation of samples into the backs of New Zealand rabbits. Subcutaneous implantation models were created in four rabbits (n = 4) weighing 2.0–2.5 kg following a protocol approved by Xi'an Medical University (NO.XYLS2019139). The rabbits were anesthetized by injection with pentobarbital sodium (20 mg/kg). The fur on the back of the rabbits was shaved and the three experimental cement disks were implanted into the dorsum of a single rabbit. After 4 weeks of implantation, bone cement implants and the surrounding tissue were harvested from the euthanized animals.

### Histomorphometric and immunohistochemistry (IHC) analyses

The Harvested samples were then washed with normal saline, fixed in 4% paraformaldehyde for 48 h and decalcified in EDTA for 4 weeks. The samples were dehydrated in a graded series of ethanol solutions, embedded into paraffin then sliced into sections with a thickness of 5 μm. The slides were stained with hematoxylin & eosin (H&E) and Masson trichrome. Images of stained slides were examined and digitized under a light microscope at 10 magnification to evaluate the osteogenic capacity. New bone was quantified through measuring the area of newly formed collagen tissue in Masson staining using ImageJ software. The expression of pro-inflammatory cytokines within the harvested tissues were detected using IHC-staining with anti-TNF-α and anti-IL-1β (Proteintech Group, China) as primary antibodies. The values of average optical density (AOD) of the IHC-stained images were measured for quantitative analysis by the ImageJ software.

### Statistical analysis

Statistic analysis was performed by using the software Statistical Package for the Social Sciences (SPSS) version 17.0. One-way analysis of variance (ANOVA) and LSD test were used to determine the significance of differences. In all statistical evaluations, *P* < 0.05 was considered as statistically significant.

## Data Availability

All the data and materials of the research are available.
